# Differences in lower extremity biomechanics, shank muscle activation and medial gastrocnemius–tendon unit behavior between novice and experienced non-rearfoot strike runners

**DOI:** 10.3389/fbioe.2025.1641666

**Published:** 2025-09-17

**Authors:** Bokai Suo, Zeyu Lu, Jichao Wang, Kaicheng Wu, Liqin Deng, Lu Li, Yunjian Zhong, Weijie Fu

**Affiliations:** ^1^ Key Laboratory of Exercise and Health Sciences, Ministry of Education, Shanghai University of Sport, Shanghai, China; ^2^ College of Physical Education, Nanchang University, Nanchang, China; ^3^ School of Intelligent Sports Engineering, Shanghai University of Sport, Shanghai, China

**Keywords:** foot strike pattern, kinematics, kinetics, electromyography, ultrasound

## Abstract

**Purpose:**

This study aimed to investigate the differences in lower extremity kinematics and kinetics, shank muscle activation, and medial gastrocnemius–tendon unit behavior between habitual rearfoot strike (RFS) runners and habitual non-rearfoot strike (NRFS) runners when adopting an NRFS pattern.

**Methods:**

Twelve male habitual RFS runners (novice NRFS runners, NN) and twelve male habitual NRFS runners (experienced NRFS runners, EN) were recruited. All participants were required to run at 9 km/h on the split-belt 3D instrumented treadmill using an NRFS pattern. 3D lower extremity kinematics and kinetics, surface electromyography (sEMG) signals of medial and lateral gastrocnemius (MG and LG), soleus (SOL), and tibialis anterior (TA), as well as dynamic ultrasound imaging of MG tendon unit behavior during running were collected synchronously. Intergroup comparisons were performed using independent samples t-tests and Mann–Whitney U tests, with Significance levels (*α*) adjusted via Bonferroni correction.

**Results:**

Compared to EN, NN exhibited significantly greater fascicle shortening lengths (NN: 1.54 ± 0.66 cm; EN: 0.94 ± 0.23 cm; *p* = 0.013) and muscle–tendon unit (MTU) shortening lengths (NN: 3.45 ± 0.51 cm; EN: 1.96 ± 0.23 cm; *p* < 0.001) of MG. No intergroup differences were observed in lower extremity kinematics, kinetics, or shank muscle activation.

**Conclusion:**

While novice and experienced NRFS runners exhibited similar kinematic, kinetic and muscle activation characteristics, the increased fascicle and muscle–tendon unit shortening lengths of medial gastrocnemius in novice NRFS runners potentially reflect reduced muscle contraction efficiency.

## 1 Introduction

Running offers multiple health benefits, and participation rates have surged in recent years (Lee et al., 2017). Current running-related research has increasingly focused on foot strike patterns, which are mainly categorized into rearfoot strike patterns (RFS) and non-rearfoot strike patterns (NRFS, i.e., forefoot/midfoot strike) (Lieberman et al., 2010). NRFS may offer several potential benefits. Compared to habitual RFS runners, NRFS runners typically exhibit shorter contact times at the same stride frequency and greater leg stiffness, which was thought to be associated with a better running economy in some studies (Anderson et al., 2020; Jaén-Carrillo et al., 2022; Lieberman et al., 2015), and achieve faster finishing times in road events (Bovalino et al., 2019). Besides, the muscle–tendon unit (MTU) of habitual NRFS runners has been reported to generate more efficient contraction and elastic return, potentially enhancing running performance (Deng et al., 2023). Additionally, NRFS adoption has been associated with a reduced first peak vertical ground reaction force (vGRF) and lower loading rate during the stance phase, suggesting an attenuated impact loading pattern, thereby potentially reducing impact-related injuries (Boyer et al., 2014). This reduction in impact force may be attributed to the biomechanical mechanism whereby NRFS converts a portion of the vertical impact energy into rotational energy of the foot around the ankle, thereby substantially reducing the effective mass during collision and mitigating cumulative bone stress, which may help prevent stress-related fractures (Boyer et al., 2014; Lieberman et al., 2010; Nigg et al., 2023).

However, the biomechanical advantages of NRFS remain debated. For example, adopting an NRFS pattern may increase biomechanical loading of the Achilles tendon and calf muscles, potentially raising the risk of tendon-related injuries and chronic overuse conditions (Rice and Patel, 2017; Kulmala et al., 2013). Moreover, evidence regarding running economy improvements is inconsistent, with some studies reporting no significant economic advantage or even increased metabolic costs when using an NRFS pattern (Gruber et al., 2013). Despite potential biomechanical advantages, Bovalino et al. (2019) reported that more than 76% of runners adopt an RFS pattern during road running events, whereas only a small percentage consistently maintain an NRFS pattern throughout an entire race. Thus, individual differences, adaptability, and long-term biomechanical adaptations must be carefully considered when evaluating the appropriateness of adopting an NRFS running pattern (Anderson et al., 2020).

On the basis of the abovementioned advantages, some habitual RFS runners exhibit intentionality toward adopting NRFS patterns. Valenzuela et al. (2015) found that habitual RFS runners can transition to NRFS running without long-term preparation. Specifically, these novice NRFS runners (novice non-rearfoot striker, NN, i.e., habitual RFS runners who acutely transition to NRFS running) exhibit similar lower extremity kinematics and kinetics to experienced NRFS runners (experienced non-rearfoot striker, EN, i.e., habitual NRFS runners). However, consider that habitual NRFS runners exhibit greater ankle muscle force-generating capacity during running due to their higher ankle joint work (Almeida et al., 2015). Therefore, although these NN runners can achieve convergence in apparent biomechanical characteristics, the immediate alteration in movement patterns may lead to muscle maladaptation (Douglas et al., 2017; Deng et al., 2024). Such maladaptation may be associated with energy utilization efficiency and tissue loading (Holt and Mayfield, 2023).

Changes in foot strike patterns are primarily driven by alterations in ankle motion, which is mainly controlled by the triceps surae (Deng et al., 2023; Swinnen et al., 2019b). The energy consumption of the triceps surae accounts for 22%–32% of the total energy expenditure during running (Swinnen et al., 2019a), and the muscle behavior and activation of the triceps surae can affect push-off efficiency and energy cost during running (Farris et al., 2016; Fletcher and MacIntosh, 2017). Therefore, investigating differences in shank muscle behavior and activation between these two habitual foot strikers when NRFS running may provide neuromuscular insights into muscle adaptation strategies and force production efficiency.

This study aimed to investigate the differences in lower extremity kinematics and kinetics, shank muscle activation, and medial gastrocnemius–tendon unit behavior between NN and EN runners when adopting an NRFS pattern. The study hypothesized that compared with EN, NN would have: 1) similar kinematic and kinetic characteristics; 2) significantly greater muscle activation during the push-off phase; and 3) NN might exhibit different behavior of the medial gastrocnemius (MG) fascicle and MTU with EN.

## 2 Methods

### 2.1 Participants

G*power (Version 3.1.9.7, Heinrich Heine University, Germany) was used to calculate the sample size. Cohen’s d was determined by the MG fascicle shortening length in different habitual foot strikers as reported by Swinnen et al. (2019b). *A priori* power analysis (α = 0.05, 1-β = 0.8, Cohen’s d = 1.256) indicated that a sample size of 11 participants per group were needed for the independent t-test. Furthermore, a *post hoc* power analysis based on the MG fascicle and shortening length observed in this study were 0.811 and 1.000, indicating adequate power for detecting the observed effect.

A total of 24 male runners were recruited for this study based on the following criteria: 1) running distance ≥20 km per week for the last 3 months; 2) absence of neurological disease, as well as no pain and injury to the lower limbs, triceps surae, and Achilles tendon, within the last 6 months; and 3) maintenance of a habitual foot strike pattern for at least 1 year (Ye et al., 2024). 12 male habitual RFS runners were recruited in the NN group, and 12 male habitual NRFS runners were recruited in the EN group. Participant information is shown in Table 1. All participants signed an informed consent form approved by the ethics committee of the local university (No. 102772021RT085).

**TABLE 1:** Participant information.

Information	NN (n = 12)	EN (n = 12)	*p*-value
Age (years)	32.50 ± 7.80	34.08 ± 11.07	0.689
Height (cm)	174.67 ± 3.73	173.00 ± 4.59	0.340
Body Weight (kg)	68.01 ± 8.94	71.33 ± 8.84	0.590
Weekly Running Distance (km)	39.88 ± 19.12	47.88 ± 27.61	0.418

Notes: novice non-rearfoot strikers (NN); experienced non-rearfoot strikers (EN).

### 2.2 Procedure

After participants arrived at the laboratory, an experienced researcher measured their height and weight. Following this, participants were asked to wear Nike Pegasus 34 shoes (forefoot stack: 20 mm; heel stack: 30 mm; heel–toe drop: 10 mm) and warmed up with habitual foot strike patterns at a self-determined speed. This shoe was chosen because its 8–10 mm heel–toe drop (HTD) represents mainstream running footwear for recreational runners, ensuring ecological validity (Malisoux et al., 2016); moreover, systematic evidence confirms that such HTD values do not significantly alter ankle/knee kinematics when foot strike patterns are standardized, thus minimizing confounding effects (Sánchez-Ramírez et al., 2023), and prior studies have successfully quantified NRFS running biomechanics using identical 10 mm HTD shoes (Besson et al., 2019; Zhang et al., 2025). To verify participants’ self-reported habitual foot strike patterns, their running motion was recorded using the slow-motion feature of a smartphone camera (Valenzuela et al., 2015).

**FIGURE 1:**
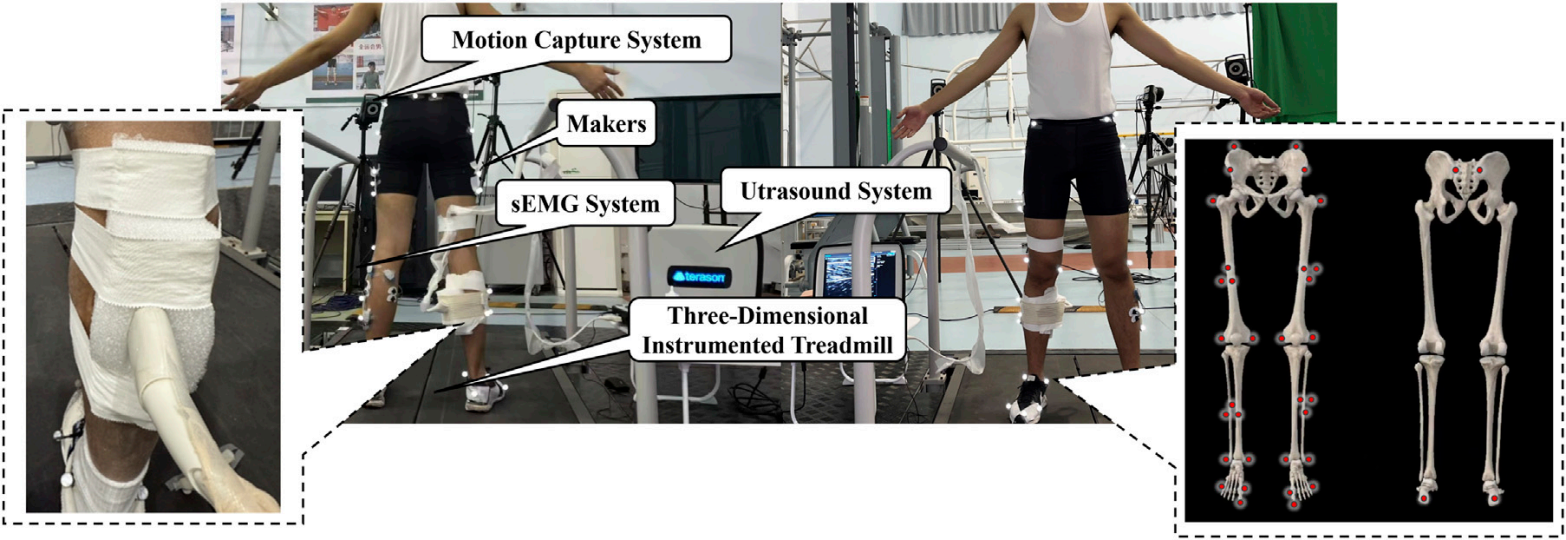
Experimental setup; probe and markers placement. Notes: surface electromyography (sEMG).

After 5 min of warm-up, infrared reflective markers were placed in the following landmarks of the participant based on the Visual3D Hybrid model: anterior and posterior superior iliac spines, midpoint of the iliac crests, greater trochanters, medial and lateral femoral epicondyles, medial and lateral malleoli, heel, 1st and 5th metatarsal heads, and 1st distal phalanx. A rigid body consisting of three non-collinear reflective markers was placed on the lateral side of the thighs and shanks. By using a self-made foam model and bandages, an ultrasonic probe (12L5A, frequency: 12 MHz) was placed at 30% of the distance between the popliteal crease and the malleolus along the longitudinal axis of the dominant leg, where the deep and superficial aponeurosis were visually confirmed to maintain parallel alignment under imaging (Figure 1) (Deng et al., 2023; Monte et al., 2020). Prior to the formal test, each participant completed an adaptive running session on the treadmill while the ultrasound probe was fixed in place. During this familiarization period, researchers evaluated the ultrasound images for quality and stability and further reinforced the probe fixation as necessary to ensure consistent image acquisition throughout the experiment. The reliability of this method was mentioned in a previous study (ICC = 0.834–0.958) (Deng et al., 2023); therefore, it is considered reliable for acquiring ultrasound images of MG behavior during dynamic running tasks.

After skin preparation, surface electromyography (sEMG) electrodes were attached to the belly of the soleus (SOL), medial and lateral gastrocnemius (MG and LG), and tibialis anterior (TA) of another leg.

Before the formal test, runners in the NN group acclimated to the treadmill with the NRFS pattern without any specific instructions. Runners in the EN group acclimated to the treadmill with their habitual foot strike pattern. Subsequently, static models were captured. In the formal test, runners ran continuously for 5 min in the NRFS pattern on the split-belt 3D instrumented treadmill (Fully Instrumented Treadmill, Bertec, United States, sampling rate: 1,000 Hz) at 9 km/h. An external custom foot switch was used to synchronize the collection of ground reaction force, marker trajectories (T040, Vicon, United Kingdom; sampling frequency: 200 Hz), sEMG signals (Ultium, Noraxon EMG, United States; sampling rate: 2000 Hz), and dynamic ultrasound imaging (uSmart 3300, TELEMED, United States; sampling rate: 22 Hz) during the final minute of running, when the runner’s movement had stabilized. A digital trigger signal from the foot switch was simultaneously sent to all acquisition systems to ensure frame-level synchronization across modalities.

### 2.3 Data processing

#### 2.3.1 Kinematics and kinetics

The kinematic and kinetic data were processed by Visual 3D (Version: 3.2.1, C-Motion, Inc., United States). The GRF data were subjected to low-pass filtering at a cut-off frequency of 50 Hz, and the marker trajectories were filtered at a low-pass cut-off frequency of 7 Hz (Zhang et al., 2021). Based on a vertical GRF threshold of 30 N, the timing of initial contact and toe-off during the gait cycle was determined (Swinnen et al., 2019b), and data from ten consecutive steps of the dominant leg were selected for analysis.

The foot strike angle, which was used to determine the foot strike pattern, was calculated as the angle between the vector from the heel marker to the first metatarsophalangeal joint marker and the anteroposterior axis in the laboratory coordinate system. It was then adjusted by subtracting the angle measured during static standing. A runner was classified as using a NRFS pattern when the foot strike angle was less than 8°, based on the cutoff proposed by Altman and Davis (2012), who identified this threshold using statistical boundaries derived from strike index classifications in shod runners. They demonstrated that a foot strike angle below 8° corresponds to a strike index greater than 33%, indicating a NRFS pattern.

The step frequency, contact time, and swing time were calculated based on the vertical GRF. When computing the loading rate, a point of interest was chosen where GRF was greater than 75% of body weight and exhibited an instantaneous loading rate of less than 15 BW/s (Futrell et al., 2018; Hafer et al., 2015). The average loading rate was calculated as the linear slope of the vGRF between 20% and 80% of the point of interest, whereas the instantaneous loading rate was defined as the maximum rate of force development observed within 20%–100% of the point of interest (Futrell et al., 2018; Hafer et al., 2015).

#### 2.3.2 sEMG

The sEMG data were filtered using a fourth-order Butterworth band-pass filter with a bandwidth of 20–450 Hz (Kovács et al., 2023). This cutoff range was selected based on established guidelines and practical recommendations for kinesiological EMG studies, which indicate that most of the EMG signal power is distributed between 20 Hz and 450 Hz, while lower or higher frequencies are more likely to contain motion artifacts and noise rather than meaningful physiological data (Konrad, 2005). Based on the timing of initial contact, toe-off, and the occurrence of peak knee flexion, the root mean square (RMS) values of the target muscles were calculated for the entire stride, stance phase, swing phase, loading phase, push-off phase, and pre-activation. Specifically, the loading phase was defined as the period from when the vGRF first exceeded 30 N after initial contact to the moment of peak knee flexion, while the push-off phase was defined as the period from peak knee flexion to the instant when vGRF dropped below 30 N at the end of stance. Pre-activation was calculated as the 50 m window preceding initial contact. These event definitions ensured consistent identification of analysis windows across participants and trials. The ankle co-activation ( ${CO}_{Ankle}$ ) is the RMS ratio of TA to the average of MG, LG, and SOL:

$${CO}_{Ankle}=\frac{{RMS}_{TA}}{\left({RMS}_{SOL}+{RMS}_{MG}+{RMS}_{\mathrm{LG}}\right)/3}$$

The sEMG envelope was then computed using a 63-m RMS window based on the methodology of Kovács et al. (2023). The sEMG signals were normalized by the mean of the peaks at each step.

#### 2.3.3 Behavior of MG and MTU

**FIGURE 2:**
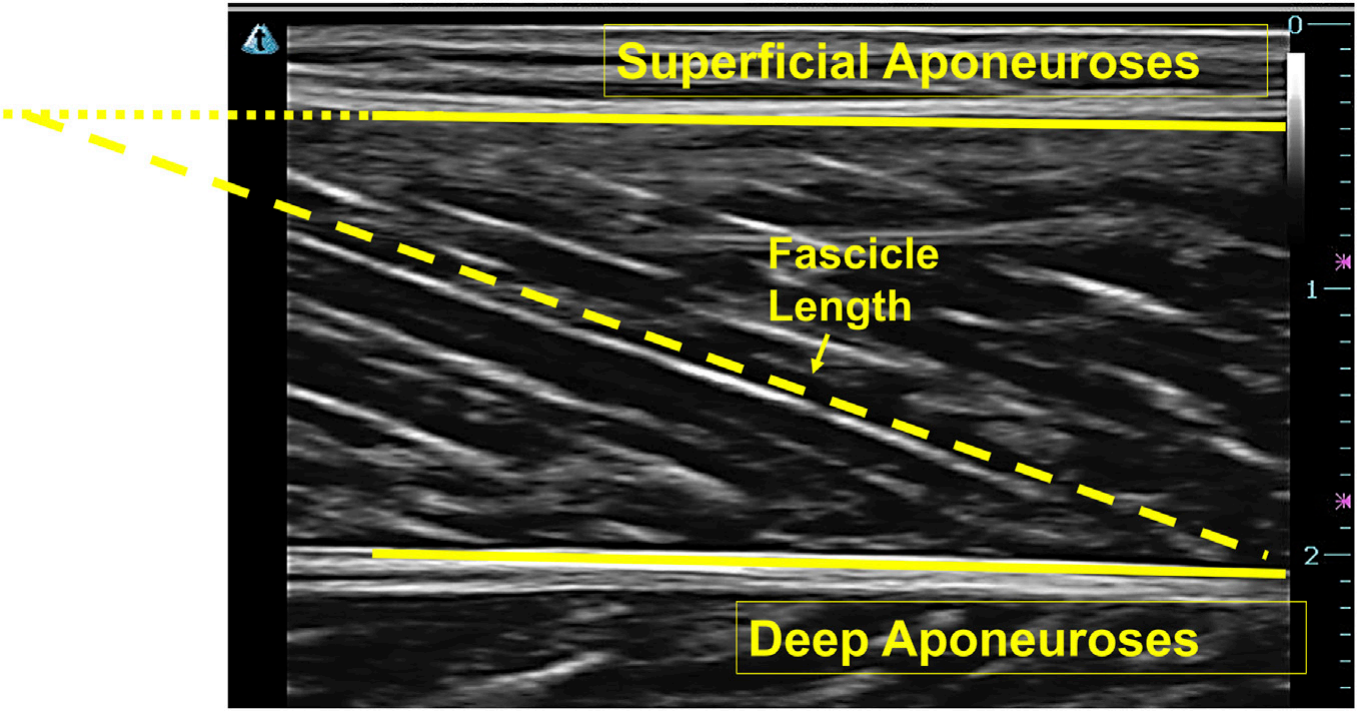
Ultrasound image of the medial gastrocnemius and the extension of the fascicle.

The Ultratrack (Version: 4.1, measurement accuracy: 0.001 m) was used to monitor ultrasound video data obtained during running to determine the fascicle length. Fascicle length was defined as the fascicle path length between superficial and deep aponeuroses. If the fascicle path was not fully visible in the image, a manual extension was made based on the orientation of superficial and deep aponeuroses and the fascicle path to estimate the missing portion (Figure 2) (Franchi et al., 2018).

Fascicle lengths at initial contact and toe-off were identified based on the GRF. The stance-phase fascicle length change was computed as the difference between these two events. Fascicle shortening length was defined as the difference between the maximum and minimum fascicle lengths during stance (Monte et al., 2020). The instantaneous shortening velocity was derived from fascicle length changes, with peak shortening velocity determined as the maximum shortening rate (Monte et al., 2020).

The peak force of the MG was calculated using the following formula:

$$F_{MG}=k\cdot \left(M/L_{AT}\right)$$

where k = 0.16 is the ratio of the MG cross-sectional area to the total triceps surae muscle cross-sectional area, as determined from population averages in healthy adults (Kubo et al., 2022), and this value represents a group-level estimate that may not capture individual anatomical variation; M is the plantar flexion moment during running; and $L_{AT}$ represents the Achilles tendon moment arm, estimated using the polynomial method proposed by Lyght et al. (2016) for calculating the triceps surae muscle–tendon moment arm, with reference to tendon imaging methods described by Rugg et al. (1990):

$$Y=-0.00591+0.0475X-0.00855X^{2}$$

where Y is the Achilles tendon moment arm, and X is the ankle angle (rad) (Deng et al., 2023).

Besides, the regression equations proposed by Hawkins and Hull (1990) were used to calculate the MTU length during running:

$$L_{MTU}=0.9+0.00214\cdot \alpha -0.00062\cdot \beta$$

Where α is the angle of ankle joint plantarflexion/dorsiflexion, and β is the angle of knee joint flexion/extension.

MTU lengths were quantified between the initial contact and toe-off events. Peak shortening velocity was derived as the stance-phase maximum of the MTU length time derivative, whereas peak stretching velocity corresponded to the minimum derivative value during stance.

### 2.4 Statistical analysis

All results were expressed as M ± SD. Intergroup comparisons were performed using SPSS (Version: 26.0, IBM Statistics, Inc., United States). The normality of the data distribution was evaluated using the Shapiro–Wilk test. If the data were normally distributed, the parameters were compared using the independent samples t-test. If the data were not normally distributed, the nonparametric Mann-Whitney U test was used. Cohen’s d and Cliff’s δ were calculated for each parameter as the effect size. In addition, an independent samples t-test from 1D Statistical Parametric Mapping (1dSPM) was used to compare sEMG signals between different groups (Pataky, 2010). To control family-wise error rates across multiple comparisons, we computed Bonferroni-adjusted the significance level (*α*) based on the number of hypotheses within each biomechanical domain: kinematics (7 comparisons); kinetics (10 comparisons); spatiotemporal parameters (3 comparisons); sEMG parameters per gait phase (5 comparisons); sEMG envelope (4 comparisons); fascicles behavior (2 comparisons); and MTU dynamics (5 comparisons).

## 3 Results

All variables were normally distributed, except for knee range of motion (ROM), peak hip extension velocity, peak joint moments (ankle, knee, and hip), peak joint powers (knee and hip) in kinematic and kinetic parameters; RMS_MG_, RMS_LG_, RMS_SOL_, CO_Ankle_ in stance phase, RMS_MG,_ RMS_TA_ in swing phase, RMS_MG_, RMS_SOL_, CO_Ankle_ in loading phase, RMS_LG_, CO_Ankle_ in push-off phase, and CO_Ankle_ in pre-activation; MTU stretching and shortening velocity.

### 3.1 Kinematics, kinetics, and spatiotemporal parameters

Based on both foot strike angle measurements and confirmation via slow-motion video recordings during the formal trials, both groups met the criteria for the defined NRFS: participants in the EN group maintained their habitual foot strike patterns, while those in the NN group successfully transitioned to NRFS running. No statistically significant differences were detected across all kinematic, kinetic, and spatiotemporal parameters between groups (Table 2).

**TABLE 2:** Kinematics, kinetics, and spatiotemporal parameters between the NN and EN groups.

Domain	Variables	NN	EN	*p*-value	Cohen’s *d*/Cliff’s *δ*
Kinematics (*ɑ* = 0.007)	Foot Strike Angle (°)	−6.64 ± 3.20	−3.08 ± 2.56	0.040	1.229
	Ankle ROM (°)	38.11 ± 5.09	33.81 ± 6.96	0.210	0.705
	Knee ROM (°)	20.94 ± 5.38	18.10 ± 2.37	0.319	0.347
	Hip ROM (°)	29.82 ± 3.51	29.17 ± 4.51	0.966	0.161
	Peak Ankle Plantarflexion Velocity (°/s)	178.47 ± 41.58	164.29 ± 44.89	0.450	0.328
	Peak Knee Extension Velocity (°/s)	93.36 ± 37.63	79.90 ± 28.74	0.356	0.402
	Peak Hip Flexion Velocity (°/s)	84.19 ± 21.65	76.63 ± 18.87	0.391	0.125
Kinetics (*ɑ* = 0.005)	Peak Ankle Plantarflexion Moment (Nm/kg)	2.91 ± 0.92	3.35 ± 0.36	0.213	0.333
	Peak Knee Extension Moment (Nm/kg)	2.26 ± 1.73	2.28 ± 1.02	0.443	0.194
	Peak Hip Extension Moment (Nm/kg)	2.33 ± 0.77	2.68 ± 0.70	0.671	0.319
	Peak Ankle Positive Power (W/kg)	13.34 ± 5.26	14.79 ± 3.87	0.630	0.314
	Peak Knee Positive Power (W/kg)	6.68 ± 4.35	6.57 ± 1.97	0.291	0.264
	Peak Hip Positive Power (W/kg)	3.66 ± 3.22	3.60 ± 1.20	0.291	0.264
	Peak Vertical GRF (BW)	2.59 ± 0.21	2.65 ± 0.34	0.702	0.212
	Average Loading Rate (BW/s)	38.42 ± 8.71	53.09 ± 20.31	0.116	0.939
	Instantaneous Loading Rate (BW/s)	62.01 ± 7.87	79.37 ± 20.03	0.007	1.141
	Leg Stiffness (BW/m)	36.11 ± 5.09	44.26 ± 7.02	0.013	1.329
Spatiotemporal Parameters (*ɑ* = 0.017)	Contact Time (s)	0.23 ± 0.03	0.22 ± 0.03	0.238	0.333
	Flight Time (s)	0.12 ± 0.02	0.11 ± 0.03	0.918	0.392
	Step Frequency	172.02 ± 10.95	179.42 ± 10.32	0.117	0.696

Notes: Significance levels (*α*) were adjusted for multiple comparisons using Bonferroni correction. Novice non-rearfoot striker (NN); experienced non-rearfoot striker (EN); range of motion (ROM); body weight (BW).

### 3.2 sEMG

**FIGURE 3:**
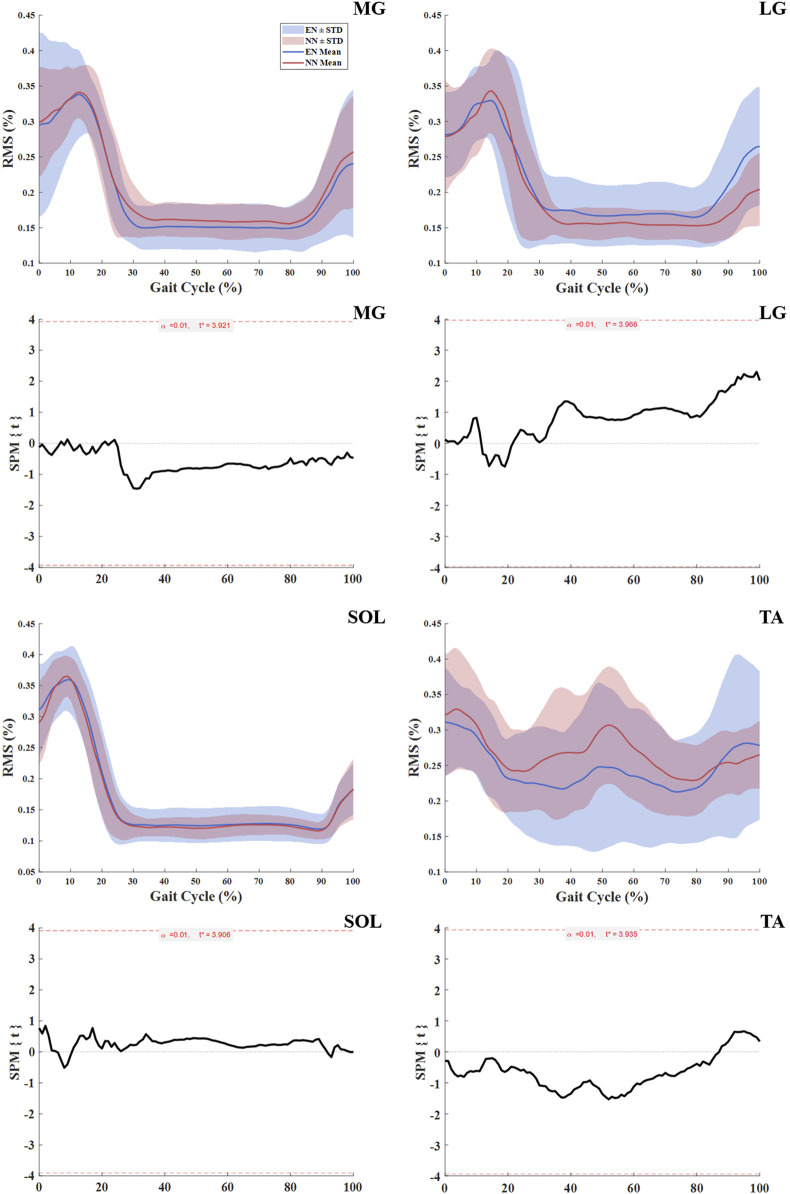
Muscle activation in NN and EN runners. Notes: Significance levels (*α*) were adjusted for multiple comparisons using Bonferroni correction. Root mean square (RMS) medial gastrocnemius (MG); lateral gastrocnemius (LG); soleus (SOL); tibialis anterior (TA); novice non-rearfoot striker (NN); experienced non-rearfoot striker (EN).

No statistically significant differences in shank muscle activation were observed between the NN and EN groups throughout the entire gait cycle (Table 3; Figure 3).

**TABLE 3:** RMS of MG, LG, SOL, TA, and ankle co-activation between the NN and EN group.

Phase	Variables	NN	EN	*p*-value	Cohen’s *d*/Cliff’s *δ*
Stride Cycle (*ɑ* = 0.010)	RMS_MG_ (%)	0.15 ± 0.02	0.15 ± 0.02	0.716	0.151
	RMS_LG_ (%)	0.15 ± 0.02	0.15 ± 0.02	0.566	0.238
	RMS_SOL_ (%)	0.14 ± 0.01	0.13 ± 0.02	0.294	0.439
	RMS_TA_ (%)	0.19 ± 0.04	0.18 ± 0.04	0.914	0.045
	CO_Ankle_ (%)	0.43 ± 0.09	0.43 ± 0.09	0.812	0.098
Stand Phase (*ɑ* = 0.010)	RMS_MG_ (%)	0.21 ± 0.07	0.23 ± 0.03	0.713	0.097
	RMS_LG_ (%)	0.23 ± 0.05	0.22 ± 0.02	0.198	0.319
	RMS_SOL_ (%)	0.23 ± 0.05	0.21 ± 0.05	0.266	0.278
	RMS_TA_ (%)	0.20 ± 0.04	0.18 ± 0.05	0.336	0.402
	CO_Ankle_ (%)	0.34 ± 0.19	0.28 ± 0.09	0.478	0.181
Swing Phase (*ɑ* = 0.010)	RMS_MG_ (%)	0.11 ± 0.03	0.11 ± 0.03	0.671	0.111
	RMS_LG_ (%)	0.10 ± 0.02	0.11 ± 0.03	0.290	0.442
	RMS_SOL_ (%)	0.08 ± 0.02	0.08 ± 0.02	0.876	0.065
	RMS_TA_ (%)	0.18 ± 0.04	0.17 ± 0.06	0.932	0.028
	CO_Ankle_ (%)	0.84 ± 0.31	0.82 ± 0.57	0.937	0.032
Loading Phase (*ɑ* = 0.010)	RMS_MG_ (%)	0.22 ± 0.10	0.26 ± 0.06	0.443	0.194
	RMS_LG_ (%)	0.23 ± 0.08	0.24 ± 0.03	0.603	0.217
	RMS_SOL_ (%)	0.27 ± 0.08	0.26 ± 0.07	0.514	0.167
	RMS_TA_ (%)	0.23 ± 0.06	0.22 ± 0.07	0.563	0.240
	CO_Ankle_ (%)	0.86 ± 1.90	0.30 ± 0.11	0.410	0.208
Push-off Phase (*ɑ* = 0.010)	RMS_MG_ (%)	0.19 ± 0.06	0.21 ± 0.04	0.341	0.397
	RMS_LG_ (%)	0.22 ± 0.05	0.20 ± 0.04	0.160	0.347
	RMS_SOL_ (%)	0.18 ± 0.04	0.16 ± 0.04	0.204	0.535
	RMS_TA_ (%)	0.16 ± 0.04	0.15 ± 0.05	0.406	0.346
	CO_Ankle_ (%)	0.31 ± 0.17	0.26 ± 0.09	0.671	0.111
Pre-activation (*ɑ* = 0.010)	RMS_MG_ (%)	0.17 ± 0.09	0.18 ± 0.07	0.776	0.118
	RMS_LG_ (%)	0.15 ± 0.05	0.18 ± 0.04	0.082	0.745
	RMS_SOL_ (%)	0.13 ± 0.06	0.14 ± 0.03	0.652	0.188
	RMS_TA_ (%)	0.17 ± 0.04	0.23 ± 0.08	0.065	0.793
	CO_Ankle_ (%)	1.21 ± 2.79	0.55 ± 0.35	0.713	0.097

Notes: Significance levels (*α*) were adjusted for multiple comparisons using Bonferroni correction. Root mean square (RMS); co-activation (CO); medial gastrocnemius (MG); lateral gastrocnemius (LG); soleus (SOL); tibialis anterior (TA); novice non-rearfoot striker (NN); experienced non-rearfoot striker (EN).

### 3.3 Behavior of fascicle and MTU

**FIGURE 4:**
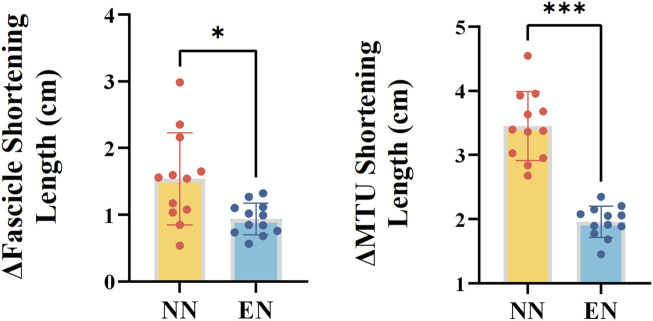
Mechanics and behavior of the MG variables with significant differences between the two groups. Notes: *: *p* < 0.05; ***: *p* < 0.001. Muscle–tendon unit (MTU); novice non-rearfoot striker (NN); experienced non-rearfoot striker (EN).

For the mechanics and behavior of the fascicle and MTU, the NN group exhibited greater fascicle and MTU shortening length of MG compared with the EN group (fascicle: *p* = 0.038, Cohen’s *d* = 1.214; MTU: *p* < 0.001, Cohen’s *d* = 3.766, Table 4; Figure 4).

**TABLE 4:** Variables of MG behavior between the NN and EN group.

Domain	Variables	NN	EN	*p*-value	Cohen's *d*/Cliff's *δ*
Fascicles Behavior (*ɑ* = 0.025)	**ΔFascicle Shortening Length (cm)**	**1.54 ± 0.66**	**0.94 ± 0.23**	**0.013**	**1.214**
	Peak Fascicle Shortening Velocity (cm/s)	16.23 ± 6.94	11.16 ± 2.70	0.040	0.963
MTU dynamics (*ɑ* = 0.010)	Peak MG Force (BW)	1.17 ± 0.19	1.07 ± 0.11	0.119	0.644
	**ΔMTU Shortening Length (cm)**	**3.45 ± 0.51**	**1.96 ± 0.23**	**< 0.001**	**3.766**
	ΔMTU Stretching Length (cm)	2.27 ± 0.51	1.85 ± 0.37	0.123	0.943
	Peak MTU Shortening Velocity (cm/s)	42.35 ± 15.1	33.45 ± 5.85	0.146	0.292
	Peak MTU Stretching Velocity (cm/s)	36.65 ± 8.03	29.33 ± 5.10	0.018	0.556

Notes: Significance levels (*α*) were adjusted for multiple comparisons using Bonferroni correction. Bold: Significant difference between NN and EN. Novice non-rearfoot striker (NN); experienced non-rearfoot striker (EN); body weight (BW); medial gastrocnemius (MG); muscle-tendon unit (MTU).

## 4 Discussion

This study investigated the biomechanics, muscle activation and behavior differences between NN and EN in the lower extremities. No significant differences were found between the NN and EN groups in terms of kinematic, kinetic and spatiotemporal parameters (Table 2); this result was aligned with our first hypotheses, which both groups of runners exhibited similar kinematic and kinetic characteristics. No significant differences in activation of the MG, LG, SOL, and TA between these two groups (Figure 3), which contradicted our second hypothesis, which runners in NN group might exhibit significantly greater muscle activation. Additionally, significant differences were observed in muscle behavior parameters: the MG fascicle shortening length and MTU shortening length was greater in the NN group than in the EN group (Table 4). These findings partly supported our third hypothesis, which runners in NN have different behavior of the MG fascicle and MTU with EN.

In this study, no significant differences were observed between two groups regarding the kinematic and kinetic parameters of the ankle, knee, and hip joints, and we found no differences in spatiotemporal parameters (Table 2). These results aligned with the findings of Valenzuela et al. (2015) that habitual RFS runners exhibit similar kinematics and kinetics when acutely adopting NRFS pattern compared with habitual NRFS runners. This might suggest that habitual RFS runners may immediately replicate the movement patterns of habitual NRFS runners, including vGRF and the kinematic and kinetic parameters of the hip, knee, and ankle, without requiring guidance or training.

No significant differences in muscle activation during the entire gait cycle were observed between the two groups of runner in this study (Table 3; Figure 3). It must be acknowledged, however, that the use of RMS values over the entire gait cycle may not capture subtle differences in the timing of muscle activation, such as onset or offset shifts, that could exist between groups. Nevertheless, the present findings still provide valuable insight. Ahn et al. (2014) reported that habitual RFS runners who acutely switch to NRFS demonstrate similar muscle activation patterns to those of habitual NRFS runners during barefoot running, which was consistent with the results of this study. This result suggests that no matter the level of a runner’s NRFS running experience, differences in their muscle activity are primarily dominated by kinematic and kinetic changes associated with the foot strike pattern. For example, the NRFS pattern requires pre-activation of the triceps surae to stabilize the ankle and cushion impact (Ahn et al., 2014), while the RFS pattern relies on TA activity to control heel strike (Ervilha et al., 2017). Previous studies could support this view: Compared to habitual RFS runners, habitual NRFS runners demonstrate lower TA activation and higher MG and LG activation during pre-activation phases (Yong et al., 2014). Notably, Kovács et al. (2023) found that when habitual RFS runners immediately adopted an NRFS pattern, they exhibited muscle activation patterns similar to those of habitual NRFS runners. This further indicates that when habitual RFS runners acutely adopt NRFS running, their neuromuscular systems could achieve convergence in lower-limb kinematics and kinetics through rapid adjustments in both the timing and amplitude of muscle activation patterns. Therefore, habitual RFS runners can shift their muscle activation patterns to resemble those of habitual NRFS runners when adopting NRFS running.

The results of this study showed that, the NN group exhibited greater fascicle shortening length during the stance phase compared with the EN group (Table 4; Figure 4). Deng et al. (2023) reported a positive correlation between foot strike angle and fascicle shortening length when runners adopt their habitual foot strike patterns. However, in this study, although the foot strike angle of NN runners was slightly lower than that of EN runners, their fascicle shortening length was significantly greater. Gonzales et al. (2019) reported that habitual NRFS runners had greater cross-sectional area and pennation angle, which could enhance the muscle’s force potential compared to habitual RFS runners. This may suggest that habitual RFS runners, owing to insufficient muscle architectural adaptations, exhibit less efficient fascicle force production during NRFS running compared to habitual NRFS runners. These results suggest that additional NRFS running exposure may be necessary for NN runners to promote architectural remodeling of the triceps surae and improve muscle contraction efficiency (Zhang et al., 2024; Deng et al., 2024).

In addition, this study also found that MTU shortening length of NN runners was significantly greater than EN runners. This finding aligned with the study of Deng et al. (2023), who reported a negative correlation between foot strike angle and MTU shortening length. From a structural perspective, the MTU mainly consists of a series combination of contractile and elastic components (Holt and Mayfield, 2023; Bohm et al., 2019). Therefore, the observed greater MTU shortening length in these runners was not only related to their greater fascicle shortening length but also potentially related to higher Achilles tendon hysteresis (Zhang et al., 2023). These mechanical properties may limit the capacity for elastic energy storage and return, forcing NN runners to rely more on active muscle shortening. Despite these differences in MTU behavior, ankle joint kinematics and kinetics were similar between groups. This suggests that NN runners may adopt a compensatory neuromechanical strategy to maintain joint-level outputs by increasing active muscle work (Douglas et al., 2017; Swinnen et al., 2019b). In contrast, EN runners may achieve similar joint mechanics through more efficient tendon elasticity and stretch–shortening cycle function (Holt and Mayfield, 2023; Held et al., 2023). Therefore, the increased MTU shortening observed in NN runners likely represents a transitional compensation early in their adaptation to NRFS running, prior to the development of tendon properties that support efficient energy recycling (Groeber et al., 2021).

This study had several limitations. First, all tests were conducted at a single, standardized running speed of 9 km/h, which may not fully represent the range of speeds observed in typical daily or recreational running. As biomechanical patterns can vary with running speed, this may limit the ecological validity of our findings, particularly for runners who habitually train at faster or slower paces. Second, the study included only male runners, limiting the generalizability of the findings. Future research should examine gender differences in muscle behavior and performance. Third, running economy was not directly assessed. and the observed differences in MG behavior and mechanics were not sufficient to fully explain variations in performance. Additional insights may be revealed through coordination variability and nonlinear dynamic analysis. Besides, fatigue was not considered in the present design. As prolonged or intense running may alter neuromuscular coordination and potentially shift foot strike patterns, future studies should incorporate fatigue protocols to assess its effect on muscle behavior and strike consistency. Finally, due to equipment limitations, MTU length was estimated using regression equations. This could lead to potential inaccuracies, particularly because the ultrasound probe was fixed over the MG muscle belly rather than directly at the muscle–tendon junction. Future studies should consider using advanced equipment capable of capturing larger dynamic ranges or directly tracking muscle–tendon junction movements to improve measurement accuracy.

## 5 Conclusion

Compared with habitual NRFS runners, habitual RFS runners who acutely transitioned to an NRFS pattern exhibited similar kinematics, kinetics, and shank muscle activation characteristics. This suggests that habitual RFS runners can spontaneously converge on biomechanical and muscle activation patterns similar to those of habitual NRFS runners when adopting NRFS running. However, the increased fascicle and muscle–tendon unit shortening lengths of medial gastrocnemius in novice NRFS strikers might reflect their reduced muscle contraction efficiency. This may potentially suggest a lack of adaptation in their muscle behavior.

## Data Availability

The raw data supporting the conclusions of this article will be made available by the authors, without undue reservation.
